# Individual Differences in Learning a Novel Discrete Motor Task

**DOI:** 10.1371/journal.pone.0112806

**Published:** 2014-11-11

**Authors:** Laura Golenia, Marina M. Schoemaker, Leonora J. Mouton, Raoul M. Bongers

**Affiliations:** University of Groningen, University Medical Center Groningen, Center for Human Movement Sciences, Groningen, The Netherlands; University Medical Center Goettingen, Germany

## Abstract

Many motor learning studies focus on average performance while it is known from everyday life experience that humans differ in their way of learning new motor tasks. This study emphasises the importance of recognizing individual differences in motor learning. We studied individual tool grasping profiles of individuals who learned to pick up objects with a novel tool, a pair of pliers. The pair of pliers was attached to the thumb and the index finger so that the tip of the thumb and the tip of the index finger were displaced to the beaks of the pair of pliers. The grasp component was manipulated by varying the location of the hinge of the pair of pliers, which resulted in different relations between beak opening and closing and finger opening and closing. The Wider Beak group had the hinge at 7 cm, the Same Beak group had the hinge at 10 cm (i.e., in the middle), and the Smaller Beak group had the hinge at 13 cm from the digits. Each group consisted of ten right-handed participants who picked up an object with one of the pairs of pliers 200 times on two subsequent days. Hand opening, plateau phase, hand closing, grasping time and maximum aperture were analyzed. To characterize individual changes over practice time, a log function was fitted on these dependent variables and the ratio of improvement was determined. Results showed that at the beginning stage of tool use learning the characteristic grasping profile consisted of three phases; hand opening, plateau phase and hand closing. Over practicing individual participants differed in the number of phases that changed, the amount of change in a phase and/or the direction of change. Moreover, with different pliers different learning paths were found. The importance of recognizing individual differences in motor learning is discussed.

## Introduction

The key interest in motor learning studies was, and often still is, to find general laws by averaging performance across several participants, as for instance illustrated by power laws of learning [1], [2]. However, showing generality in learning does not inform us about possible individual differences in motor learning between participants. Throughout the last decades, the few motor learning studies that did examine the nature of individual differences, have shown evidence of the importance of individual differences for both theoretical and practical aspects of motor learning e.g. [3]–[8]. To provide further evidence of individual differences in motor learning, the current study addresses individual differences in performance over time when learning a novel discrete motor task. The performance curve along which one individual evolves over time is what we define as the individual learning path. Individual learning paths are studied by examining grasping profiles over practicing to grasp an object with a novel tool, a pair of pliers.

Individual differences have been emphasized in developmental studies across different tasks and movements e.g. [9]–[12]. Developmental studies regarding the development of goal-directed reaching showed that individual differences are present very early in development [11], [12]. It was shown that infants differed in the timing of reach onset and the transition to stable periods when learning to reach during the first year [11], [12]. Considering that individual differences are already found during development, developmental literature can be taken as an inspiring model and as starting point for adult motor learning studies. The dynamical system framework has influenced the field of developmental studies substantially, hence, one can also look at individual differences in adult motor learning from this perspective. This framework proposes that movements are produced from the interactions among person, task and environment e.g. [13], [14]. Properties of the sub-systems making up the person, the task, or the environment determine the result of these interactions. Zanone & Kelso [15], Kelso & Zanaone [16] and a more recent study by Kostubiec et al. [7] focused on adult motor learning of rhythmic tasks by examining learning of new relative phase relations between two fingers that are rhythmically moved. It was shown that individual differences reflect differences in the individuals' intrinsic dynamics, thus learning of rhythmically motor tasks occurs on the background of pre-existing repertoires of the individual learner. The current study on individual differences of adult motor learning was inspired by both developmental studies and studies in adults from a dynamical system framework. However, in the current study a new aspect of individual differences is examined: individual differences in learning a novel discrete task instead of a novel rhythmic task. Because of this, the methodological techniques used within the dynamical system framework are not used in the current study.

Some earlier studies [3]–[6], [8] although not from a dynamical systems approach, did emphasize individual differences in motor learning. However, these studies did not examine how individual performance evolves over practicing because performance was only analysed at discrete moments in time; either at the beginning [6], at the end [3], [5] or at the beginning and towards the end of learning [4], [8]. For example, a study analysing the beginning stage of learning by King et al. [6] examined how individuals minimize a performance score, composed of spatial error and movement time, in a star tracing task. Results showed that different groups could be distinguished, one reducing spatial error, one reducing movement time and another one reducing both variables [6]. Cesqui et al. [5], using experts who were able to show consistent behaviour in an unconstrained one-handed ball catching task, showed that also at the end stage of learning different ways of catching a ball can be observed. Although these papers pointed to differences between individuals they did not address how the performance of one individual evolves over practicing, thus individual learning paths were not analysed.

As the present study aims at revealing individual learning paths in a discrete task, we chose a goal-directed action with a novel tool. This choice was based on the following reasons: First of all, when performing goal-directed actions with a novel tool the movements of the body need to be transformed to movements of the new end-effector, the tool. These transformations are often complex [17]–[19] and have to be learned. Secondly, studies about motor learning of goal-directed actions with a tool [3], [4] pointed at the existence of individual differences. That is, Bouwsema et al. [4] indicated that participants who learned to control hand opening of a prosthetic device differed in their learning capacity. Biryukova & Bril [3] showed that expert stone knappers (detaching stone flakes) differed in the amount of kinetic energy transmitted to the stone and in the kinematic patterns of the arm. Again, these two studies did not analyse learning paths. Importantly, both studies demonstrate the suitability of studying individual differences in motor learning by means of a task in which participants have to learn to perform goal directed movements with a novel tool.

The tool that was used in the current study is a pair of pliers that is usually not used in daily living, assuring novelty of the task. The pair of pliers was attached to the thumb and the index finger so that the tip of the thumb and the tip of the index finger were displaced to the beaks of the pair of pliers. This is a tool that comes very close to a functional displacement of the tip of the thumb and index finger to the tip of the tool. In order to pick up an object with this pair of pliers, participants had to shape the aperture of the beaks of the tool as they moved the tool towards the object to be grasped. Thus, grasping an object with this pair of pliers required the participants to learn to coordinate hand opening and hand closing, which together make up the grasping profile.

When grasping an object with the natural hand using a pincer grip, thus without a tool, opening of the digits up to a maximum is usually immediately followed by closing of the digits around the object [20], [21]. Therefore, most often a single peak is found in the natural grasping profile [22]. In tool grasping on the other hand, a plateau in the aperture profile is very consistently seen [4], [23]–[27]. Bongers [23] and Gentilucci et al. [26] both reported the presence of a plateau phase when grasping with pairs of pliers even though the pairs of pliers that were used were very different in how they were held and how they transformed the movements of the fingers to the movement of the new end-effectors. Also during prosthetic use a plateau phase was reported in the hand aperture; when using a body-powered prosthesis [27] and when using myo-electric hands [4], [24], [25]. This suggests that the characteristic grasping profile of the beginning stage of tool use learning consists of three phases; hand opening, plateau phase, and hand closing. Interestingly, Bouwsema et al. [24] showed that prosthesis users who are more skilled in using their prostheses have a shorter duration of the plateau phase than prosthesis users who are less skilled. Moreover, Bouwsema et al. [25] revealed that the plateau phase shortened over learning to grasp an object with a prosthesis, suggesting that the grasp profile changes over learning. Here, we study whether there are differences between individuals in the number of phases of tool grasping that change throughout practicing, in the directions of change and in the magnitude of change in these phases, aiming to reveal differences in individual learning paths.

To get a better understanding of individual learning paths, we manipulated the grasp component by varying the location of the hinge of the pair of pliers. Varying the hinge location over the handles while keeping the length of the handles the same altered the aperture ratio between digits and beaks, which may have an impact on the grasping profile and therefore on the individual learning paths. Summarizing, the importance of emphasizing individual differences has been shown in developmental studies and in studies conducted from a dynamical system framework. To get a better understanding of individual differences in motor learning, the current study focused on individual differences in a novel discrete task. The aim of the current study was therefore to examine individual differences in how participants learn to use a novel pair of pliers when objects have to be picked up. The two key questions addressed in the current study are 1) how the different phases of tool grasping (hand opening, plateau phase and hand closing) evolve per individual throughout practicing and 2) whether the use of different pliers is learned differently.

## Method

### Participants

Thirty right-handed participants were semi-random distributed over three groups of ten (in each group 5 males and 5 females; age 21.1±1.68 year). Each participant had no prior experience using the particular pairs of pliers that were used in the current study. The participants had no neurological diseases, recent injuries or musculoskeletal problems in the neck, shoulder, arm or hand regions, and had normal or corrected to normal visual sight. Those criteria were verified by self-reports of the participants. The participants received verbal and written descriptions of all procedures and signed an informed consent before the experiment started.

The ethics committee of the Center for Human Movement Sciences, University Medical Center Groningen approved the study that was conducted according to the principles expressed in the Declaration of Helsinki.

### Material and apparatus

**Figure 1:**
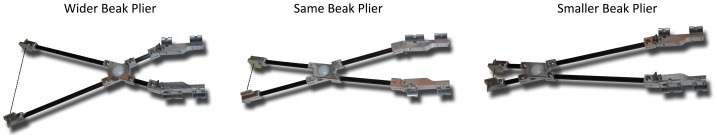
The three pairs of pliers. Note that for each pair of pliers depicted the opening at the digit side (i.e., right side) is kept the same in each picture.

Three different pairs of pliers were tested, all with a length of 20 cm. The pairs of pliers differed in the location of the hinge (Figure 1). The first group (Wider Beak group) executed the task with the Wider Beak pair of pliers with the hinge placed 7 cm away from the digits resulting in the beak opening wider than the opening of the digits. The second group (Same Beak group) used the Same Beak pair of pliers in which the hinge was placed in the middle, 10 cm away from the digits, resulting in the beak opening to be the same as the opening of the digits. The third group (Smaller Beak group) used the Smaller Beak pair of pliers with the hinge located 13 cm away from the digits causing the beak opening to be smaller than digit opening. 3D trajectories were registered with one Optotrak 3020 system sensor (Northern Digital, Waterloo, Canada), at a sampling frequency of 100 Hz. Six markers were used, two markers were attached to the tips of the pairs of pliers, two markers on the legs near the digits, and two markers on the digits themselves (index finger and thumb). For the current study, only the markers on the tips of the pair of pliers were used for analyses.

The task was performed at a table, in which a large television screen (Panasonic, 62*111 cm) was horizontally mounted and on which the starting location and object location was indicated. These locations were 30 cm apart in the anterior-posterior direction. The object that had to be picked up was a grey wooden cylinder (diameter 3 cm, height 3.5 cm) [28]–[30].

### Procedure

Participants were asked to sit comfortably in a chair in front of the table, in such a way that the start and object location were aligned with the shoulder, parallel to the sagittal plane. One leg of the pliers was attached to the thumb and one to the index finger, using elastic bands. In all trials, participants started the task with the beaks and digits closed. Participants initiated the movement following a ‘ready signal’ of the experimenter. They were instructed to reach with the pair of pliers to the object as rapidly and accurately as possible, lift it up approximately 10 cm, put it down and hold on to it until the TV screen would turn black (the TV screen turned black after 3 s). Then participants let go of the object and returned the beak to the starting location for the next trial to start. It was chosen to let the participants pick up the object because it is a quite regular procedure in prehension studies [26], [31] as it mimics the manipulation of the to be grasped object.

### Design

The study was performed in two sessions that were conducted on two subsequent days. In each session, participants picked up the object with the pair of pliers 100 times, thus 200 times in total.

### Data analysis

The trajectories of the tips of the pair of pliers were analyzed in Matlab (MathWorks; Natick, Massachusetts) using customized programs. Hand aperture was defined as the three-dimensional distance between the two markers on the beaks. Aperture velocity was computed with a three point difference algorithm. The total grasp time as well as the times used for the different movement phases (hand opening, plateau phase, and hand closing) were distinguished. To determine the duration of these phases, a backward and forward search was performed from the maximum (for hand opening) and minimum (for hand closing) in the grasp velocity profile until a threshold of 3 cm/s and −3 cm/s, respectively. The points closest to and above threshold for opening and below threshold for closing were taken as the beginning and end of the phases. Thus, hand opening was defined as the time between the start of the hand opening and the end of hand opening; hand closing was defined as the time between the start of hand closing and the end of hand closing. The period from the end of hand opening to the start of hand closing was defined as the plateau phase. Maximum aperture was computed as the maximum in the grasp component.

Changes over time in in the variables grasp time, hand opening, plateau phase, hand closing and maximum aperture were analyzed and characterized on an individual level by using a set of statistical markers. The so-called ratio of improvement (E/B) and the R^2^ of a logarithmic fit (R^2^) of the practice trials were employed. These two variables were computed for each dependent variable (see later) and separately for each individual participant. First, the ratio of improvement of the different dependent variables was calculated using the mean of the first 15 trials of session 1 as begin value, and the mean of the last 15 trials of session 2 as end value of the relevant variables (E/B). The ratio of improvement is therefore a statistical marker that can be considered as a percentage-changed measure as it indicates the amount of change over practicing. In order to determine the consistency of the change over practicing, a second statistical marker was calculated; the R^2^. To determine the value of R^2^, for each of the dependent variables the learning rate (Equation 1) was fitted to the series averaged over blocks of five trials. The equation used was based on Newell et al. [20]:
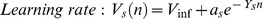
(1)Where *V_inf_* represents the asymptotic target value, *a* relates to the initial performance value and *γ* represents the slope of the function representing the learning rate. Its parameters were determined using the fminsearch function in Matlab. R^2^ was then calculated with linear regression in SPSS.

### Qualitative analysis

A three step procedure was applied to examine the individual data. First, for each individual participant both the ratio of improvement and the R^2^ were calculated for all five dependent variables, i.e. maximum aperture, grasp time, hand opening, plateau phase, and hand closing. Second, criterion values for the R^2^ and the ratio of improvement were chosen to determine which participants showed a change in either of the dependent variables. The criterion values were determined independently by three different researchers. The three researchers independently perused the learning paths of individual participants visually and compared them first with the corresponding ratio of improvement. The focus was set at distinguishing individuals with a ratio of improvement that indicated prolongation of the grasping times over practicing (i.e. E/B>1), from individuals with a decrease in the grasping times (i.e. E/B<1), and from individuals showing no changes in grasping times over practicing (i.e. E/B = 1). Then, the R^2^ was also included and compared to the learning paths and the ratio of improvement. Based on this comparison, criterion values for the R^2^ and the ratio of improvement were chosen by each researcher. After consensus of the three researchers about the criterion values; the criterion value of R^2^ was set at 0.4 meaning that a R^2^ larger than 0.4 indicated that changes had occurred during the 200 trials. A ratio of improvement smaller than 0.65 or larger than 1.35 was taken as boundaries to state that changes had occurred (i.e., E/B<0.65 or E/B>1.35 indicate a change). Thus, the combination of a low value of R^2^ and a ratio of improvement near one indicated that no changes had occurred. The third step in the procedure was to determine whether a participant showed changes over practicing in each dependent variable by scoring a ‘change’ if both scores for each dependent variable met the criterion value and scoring a ‘no change’ if no or only one criterion value was met.

The term ‘practicing’ was used for repeating the task over the days and the term ‘learning’ was used for changes in behavior over time. Therefore, it can be said that changes when repeating the task over the days, reflected practicing, and a ‘change’ in both the ratio of improvement and the R^2^ was an indication of learning, as behavior changed over time.

### Quantitative analysis

All statistical analyses were executed using SPSS software (IBM, Armonk, New York). To determine whether there is a difference between pairs of pliers in maximum aperture, a between-subject one-way ANOVA was conducted. A repeated measures multivariate ANOVA was performed with the R^2^ as dependent variable, and grasping phases (hand opening, plateau phase, hand closing) as within-subject variable and pairs of pliers as between-subject variable (Wider Beak, Same Beak, Smaller Beak). Post-hoc tests were performed with Bonferonni correction. The same analysis was performed for the ratio of improvement. The level of significance was set at α≤0.05.

## Results

In total, 6000 trials were measured in the current study. 228 unusable trials were removed from the dataset because markers were invisible so that one or more of the variables could not be determined, or when the task was executed incorrectly, for instance when the object was dropped. This left 5772 trials that were used for analysis. Out of the trials where the object has been dropped, 67.4% occurred in the first 15 trials, indicating that participants failed to perform the task. This shows that the task cannot be performed right away, but has to be learned.

### Grasping patterns

**Figure 2:**
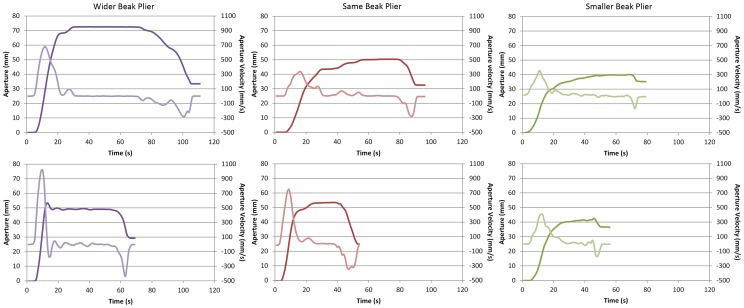
Grasping patterns of three different individuals each using a different pairs of pliers. The top row shows grasping patterns from beginning of the first practice sessions and the bottom row from the end of the second practice session. The aperture profile is the dark line and the aperture velocity is the light line.

The grasping pattern of all participants was characterized by the hand opening to a certain aperture close to the maximum (hand opening), minimal changes of that aperture for a certain time (plateau phase), followed by the closure around the object (hand closing) (Figure 2). During the plateau phase the hand opening velocity stayed around zero what can clearly be seen in Figure 2. Three aspects of these grasping patterns stood out: First, the grasping pattern of all three pairs of pliers showed a pronounced plateau phase. Importantly, this plateau phase was observable in the aperture profile of the first trials in all participants. Second, the length of the three phases (hand opening, plateau, hand closing) changed over practice in most of the participants. And third, the maximum aperture of the grasp differed for the different pairs of pliers; as expected the maximum aperture was widest for the Wider Beak plier and smallest for the Smaller Beak plier for most of the participants (Figure 2). The mean maximum aperture for the Same Beak plier was 54.01 (±11.59), for the Wider Beak plier 61.92 (±19.69) and for the Smaller Beak plier 38.31 (±5.68). Results of the one-way ANOVA showed that the maximum aperture of the three pairs of pliers was significantly different (F(2,27) = 13.52, p<0.001). Post-hoc tests showed differences between the Same Beak plier (p<0.01) and the Smaller Beak plier as well as between the Wider Beak plier and the Smaller Beak plier (p<0.01).

### Individual learning path

**Figure 3:**
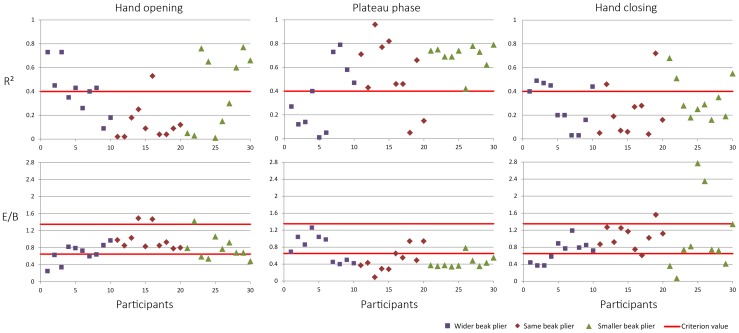
Data distribution of the R^2^ and the ratio of improvement of all participants. The R^2^ and the ratio of improvement are plotted for all participants for the variables hand opening, plateau phase and hand closing. The Wider Beak group is indicated by blue points, the Same Beak group by red points and the Smaller Beak group by green points. The red lines indicate the criterion values determined for the corresponding variable.

Figure 3 shows the data distribution of the ratio of improvement and the R^2^ for all participants for the variables hand opening, plateau phase and hand closing (data of Table 1). As can be seen for all variables the ratio of improvement and the R^2^ vary gradual over participants; there are no strong natural cuts in the data distributions. This indicates that there are a large variety of individual learning paths and that it is hard to distinguish clear-cut learning strategies. Therefore, to distinguish learning path categories the combination of the plots of the individual change over practicing, together with the values for the ratio of improvement and the R^2^ are used to determine the criterion values as described in the methods. These criterion values were chosen and shown in the figure with the red lines. For the R^2^ the red line indicates that the participants above the line were showing changes over practicing and for the ratio of improvement they indicate that the participants above the high line and below the low line were showing changes. Note that eventually both criteria must be complied for that variable of the participant to be marked as a change. The different learning paths that we found in this way are described in detail in the next paragraph.

**Table 1:** R^2^ and ratio of improvement (E/B) for all participants and dependent variables maximum aperture, grasp time, hand opening, plateau phase and hand closing.

Plier	Partic.	Aperture		Grasp		Open		Plateau		Close	
		E/B	R^2^	E/B	R^2^	E/B	R^2^	E/B	R^2^	E/B	R^2^
Wider	1	0.97	0.03	0.54	0.72	0.25	0.73	0.69	0.27	0.44	0.41
	2	0.61	0.27	0.75	0.40	0.63	0.45	1.04	0.12	0.37	0.49
	3	0.93	0.24	0.62	0.61	0.34	0.73	0.86	0.14	0.37	0.47
	4	0.75	0.59	1.04	0.00	0.82	0.35	1.26	0.40	0.58	0.45
	5	0.98	0.05	0.92	0.14	0.79	0.43	1.04	0.01	0.89	0.20
	6	0.77	0.67	0.84	0.26	0.73	0.26	0.98	0.05	0.77	0.20
	7	1.21	0.27	0.58	0.85	0.60	0.40	0.45	0.73	1.19	0.03
	8	1.33	0.52	0.52	0.83	0.64	0.43	0.40	0.79	0.79	0.03
	9	1.16	0.48	0.62	0.72	0.86	0.09	0.50	0.58	0.85	0.16
	10	0.89	0.44	0.57	0.74	0.97	0.18	0.42	0.47	0.72	0.44
Same	11	0.90	0.43	0.60	0.76	0.98	0.02	0.37	0.71	0.87	0.05
	12	1.38	0.77	0.72	0.06	0.85	0.02	0.43	0.43	1.27	0.46
	13	1.19	0.40	0.54	0.89	1.03	0.18	0.09	0.96	0.92	0.19
	14	1.13	0.38	0.59	0.78	1.49	0.25	0.29	0.77	1.25	0.07
	15	1.16	0.39	0.53	0.82	0.83	0.09	0.28	0.82	1.17	0.06
	16	0.97	0.02	0.89	0.30	1.47	0.53	0.65	0.46	0.75	0.27
	17	0.76	0.69	0.64	0.82	0.85	0.04	0.55	0.46	0.61	0.28
	18	0.88	0.28	0.95	0.31	0.93	0.04	0.94	0.05	1.02	0.04
	19	1.33	0.71	0.69	0.42	0.78	0.09	0.49	0.66	1.56	0.72
	20	0.88	0.04	0.91	0.01	0.80	0.12	0.94	0.15	1.12	0.16
Smaller	21	0.85	0.77	0.50	0.72	0.79	0.05	0.37	0.74	0.36	0.68
	22	0.78	0.63	0.53	0.66	1.42	0.03	0.35	0.75	1.27	0.51
	23	0.99	0.06	0.46	0.86	0.59	0.76	0.37	0.69	0.74	0.28
	24	0.90	0.37	0.44	0.81	0.54	0.65	0.34	0.69	0.82	0.18
	25	1.02	0.15	0.52	0.80	1.06	0.01	0.36	0.74	2.77	0.25
	26	0.92	0.22	0.80	0.43	0.77	0.15	0.78	0.42	3.35	0.29
	27	0.98	0.14	0.61	0.83	0.92	0.30	0.48	0.78	0.74	0.16
	28	1.05	0.21	0.48	0.80	0.68	0.60	0.35	0.73	0.72	0.35
	29	0.97	0.04	0.52	0.71	0.68	0.77	0.43	0.62	0.41	0.19
	30	1.22	0.69	0.57	0.87	0.48	0.66	0.55	0.79	1.34	0.55

**Figure 4:**
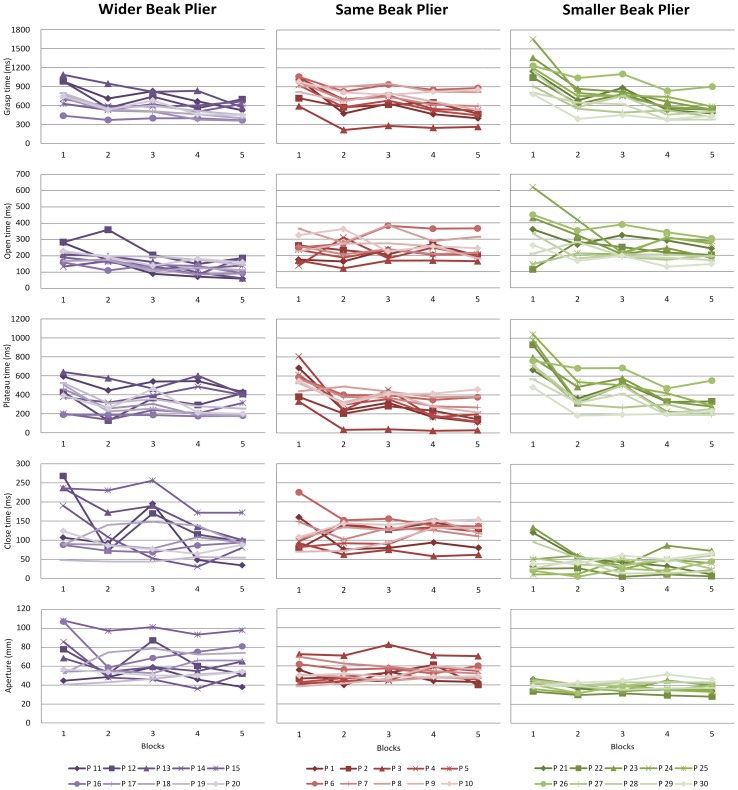
Individual learning paths. Changes over practice trials in hand opening, plateau phase, hand closing, and in aperture for all participants (10 participants in total for each pair of pliers). Blocks of five trials are presented; Block 1: Trial 1–5, Block 2: Trial 51–55, Block 3: Trial 101–105, Block 4: Trial 151–155, Block 5: Trial 195–200. Note that these are not the same blocks of trials that were used in the analyses.

**Figure 5:**
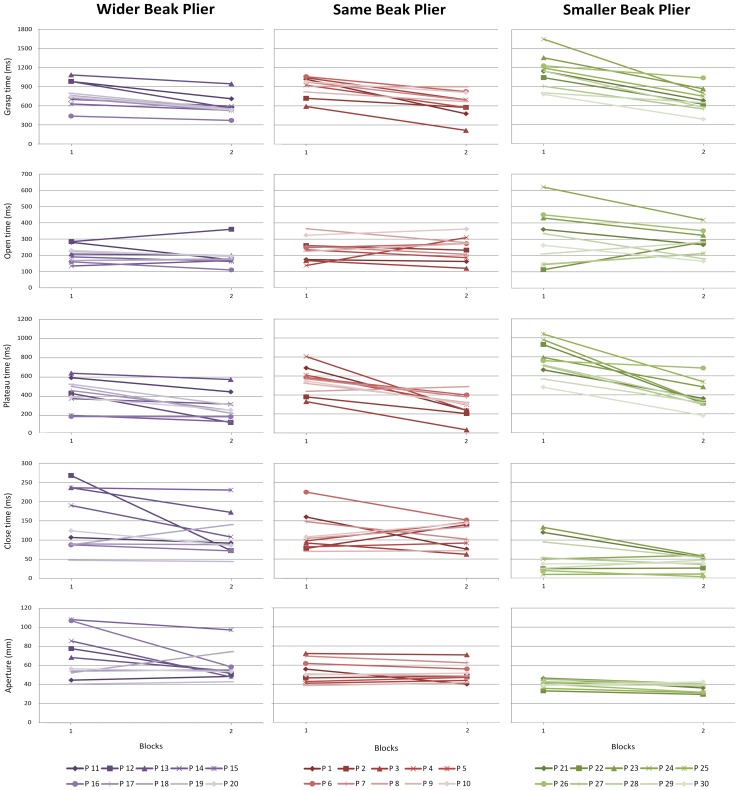
Individual changes in learning paths over the first trials of practice day 1. Changes over practice trials in hand opening, plateau phase, hand closing, and in aperture for all participants (10 participants in total for each pair of pliers) shown for block 1 (trial 1–5) and for block 2 (trials 51–55).

There were substantial individual differences in learning paths: in number of phase that changed, in magnitude of change per variable and in direction of change, which can be seen in Figure 4 and 5 as well as in Tables 1 and 2. Concerning the number of phases that were changed over practicing, results revealed that twenty-five out of the thirty participants showed a change in at least one of the phases (Table 2). The remaining five participants did not show changes in any of the phases, indicating that when learning to use a novel pair of pliers learners and non-learners could be identified. Out of the twenty-five learning participants, twenty-one revealed changes in the duration of the plateau phase, whereby thirteen of these participants only showed changes in plateau phase but no changes in other variables, and six participants showed both changes in plateau phase as well as in hand opening and/or hand closing. Four out of the twenty-five participants merely revealed changes in hand opening and/or closing time. Thus, participants differed in the number of phases that changed over learning.

**Table 2:** Assessment of changes in duration of hand opening, plateau phase and/or hand closing over learning.

Changes in hand opening	Changes in plateau phase	Changes in hand closing	Total number participants	Number participants Wider plier	Number participants Same plier	Number participants Smaller plier
−	**+**	**−**	13	2	6	5
+	**+**	**−**	6	2	1	3
−	**+**	**+**	2	0	1	1
+	**−**	**+**	3	3	0	0
**−**	**−**	**+**	1	1	0	0
−	**−**	**−**	5	2	2	1

Notes: A ‘change’ (indicated with +) or ‘no change’ (indicated with −) in the duration of hand opening, hand closing and plateau phase were scored based on the criteria of R^2^ and the ratio of improvement; both criteria must be complied.

Regarding the magnitude of change, Figure 4 and 5 show that participants differed in the magnitude of change over practicing in the variables hand opening, plateau phase, and hand closing. This is evidenced by the values of R^2^ and the ratios of improvement (Table 1). In understanding and interpreting the numbers presented in Table 1, remember that the closer the value of R^2^ to one and the further away the ratio of improvement to one, the higher the magnitude of change. To depict one example, participant 11 who used the Same Beak plier showed a low magnitude of change in hand opening and closing but showed a high magnitude of change in plateau phase. This is demonstrated by ratios of improvement near one (E/B = 0.98 and E/B = 0.87) and low R^2^'s (0.02 and 0.05) for hand opening and closing and a low ratio of improvement (E/B = 0.37) and a high R^2^ (0.71) for plateau phase (Table 1).

Finally, the direction of change over learning differed between participants. The duration of the phases could either increase (E/B>1) or decrease (E/B<1). The majority of the participants showed a decrease of the duration of the phases, especially in the plateau phase. An increase in the duration occurred more often in hand opening and closing.

In sum, individuals differed in the number of phases that changed, in magnitude of change per phase, and in the direction of change showing that there are strong individual differences in the learning paths.

### Aperture

About 93% of the participants did not adjust maximum aperture over practicing which indicates that differences within the grasping phases are not due to the size of the aperture to which the hand opened (Figure 4 and 5).

### Differences between pliers

The qualitative analysis of the individual data revealed that the different pairs of pliers showed different learning paths. The differences between the pliers became particularly clear in the number of phases that changed over learning. With two pliers, the Same Beak and the Smaller Beak, most of the participants changed the duration of the plateau phase over learning, whereas for the Wider Beak plier more often the duration of hand opening and hand closing changed. In the Same Beak group all participants who did show changes over learning revealed changes in the plateau phase over practicing (Table 1). Two out of the seven participants who showed changes in plateau phase also showed changes in hand opening or hand closing. All participants in the Smaller Beak group also revealed changes in plateau phase and three participants also showed changes in hand opening in addition to plateau phase. In the Wider Beak group only four participants changed plateau phase. Changes in hand opening and hand closing over learning occurred more often in the Wider Beak group (Table 1).

Results of the repeated measures ANOVA showed a main effect of plier for the dependent variable R^2^ (F (2,27) = 5.85, p<0.01). The mean R^2^ for hand opening for the Wider Beak plier was 0.41 (±0.21), for the Same Beak plier 0.14 (±0.16) and for the Smaller Beak plier 0.40 (±0.31). For the Wider Beak plier the mean R^2^ for plateau phase was 0.36 (±0.28), for the Same Beak plier 0.55 (±0.29) and for the Smaller Beak plier 0.70 (±0.11).The mean R^2^ for hand closing for the Wider Beak plier was 0.29 (±0.18), for the Same Beak plier 0.23 (±0.21) and for the Smaller Beak plier 0.34 (±0.18). Post-hoc tests showed differences between the Same Beak plier and the Wider Beak plier (p<0.01). The R^2^ differed for the grasping phases (F (2,26) = 8.75, p = 0.01). The interaction between these effects was not significant. The ratio of improvement showed a main effect of grasping phase (F (2,26) = 9.19, p = 0.01) but no significant differences between pliers. The mean ratio of improvement for hand opening for the Wider Beak plier was 0.66 (±0.23), for the Same Beak plier 1.00 (±0.26) and for the Smaller Beak plier 0.79 (±0.28). For the Wider Beak plier the mean ratio of improvement for plateau phase was 0.76 (±0.31), for the Same Beak plier 0.50 (±0.28) and for the Smaller Beak plier 0.44 (±0.14).The mean ratio of improvement for hand closing for the Wider Beak plier was 0.69 (±0.26), for the Same Beak plier 1.05 (±0.27) and for the Smaller Beak plier 1.13 (±1.07). The two main effects interacted (F (4,54) = 3.53, p<0.05), showing that the ratio of improvement was the same for the three phases for the Wider Beak plier but the ratio differed over the phases for the Same Beak and the Smaller Beak pliers.

## Discussion

This study demonstrated that individual differences should be taken into account when studying motor learning. We investigated changes in individual grasping profiles when practicing to pick up an object with a novel pair of pliers, a novel discrete motor task. The main findings of the current study can be summarized as follows: (a) individuals differed in their learning path, (b) the changes over learning to use a pair of pliers showed up most prominently in changes of the plateau phase, and (c) different pliers with different transformation rules showed different learning processes. Our approach of focusing on individual differences sets us apart from most of the motor learning studies that average across participants and thereby neglect individual differences resulting in possible inaccurate descriptions of practice related changes. We captured individual learning paths that may have been masked when employing averaging techniques in search for a general principle of learning. How recognizing individual differences in the learning process can contribute to the understanding of motor learning will be discussed in the following.

The key finding of the current study was that individuals revealed different learning paths. In particular, the tool grasping profile consisted of hand opening, plateau phase and hand closing and during learning, these phases changed in a different way per individual. Participants differed in the number of phases that changed, in the amount of change in each of the phases and in the direction of the change resulting in different individual learning paths. To our knowledge, individual learning paths have not yet been studied before this explicit in a discrete motor task and hence no methods were available to characterize the changes over learning of individual participants. As mentioned in the introduction, the dynamical system framework e.g. [13], [14] and several developmental studies e.g. [9]–[12] have emphasized individual differences. Because a discrete task was examined in the current study and not a rhythmical task, as is usually done in studies conducted within a dynamical system framework, the methods often used within this framework could not be applied in the current study. Therefore, the development of new methodological techniques was required. In the current study new potential techniques were introduced to analyze individual differences in a discrete task. We proposed to use the R^2^ of a logarithmic fit and the so called ratio of improvement, forming a combined measure to characterize individual learning paths. Due to the novelty of the current approach the criteria used to determine whether a change in behavior is scored as a change, were based solely on the current dataset. Using this combination of markers in future studies, examining different tasks, might further inform about the generality of the settings of the criteria we used. A limitation of the method used is that the criteria used were inspired by the current dataset and therefore might be difficult to generalize. Moreover, although we distinguished different categories the data varied gradually.

Studies examining individual differences in a rhythmical task [7], [15], [16], revealed groups who learned in two different ways: They demonstrate that participants who could at the outset of the study perform only two relative phase patterns in a stable manner showed different learning routes than participants who could perform more than two stable patterns at the outset. As mentioned before, we did not use the methods from the dynamical systems framework when investigating individual differences. However, when comparing the current study to the studies coming from this framework, it becomes clear that there is a large difference in the number of different learning groups: six different learning groups were found in the current study whereas Kostrubiec et al. [7] found only two. A possible explanation for this could be the difference between tasks. However, finding too many learning groups could bear a challenge as it becomes difficult to find general principles of motor learning. The challenge in the future will be to find a way to characterise individual differences in a structured way and to better understand why they occur.

A promising approach to perception-action learning that is helpful in understanding individual differences is the direct learning approach advocated by Jacobs and Michaels [32]–[34]. This view on learning is developed within the conceptual framework of the ecological approach, which has longstanding ties with the dynamical systems framework to action [35]–[38]. The direct learning approach aims to explain how people change from using less useful variables to more useful variables over learning a perception-action task. Therefore, the learning behaviour of an individual is portrayed in an information space. An information space has on each axis a different source of perception-action information an individual can use to perform a certain task. Each position in that space represents the informational variable, which is a combination of the informational sources, used by the individual in a specific moment in time. In this space, learning can be seen as a path representing the sequence of variables an individual exploits during learning the task. In this direct learning approach individual differences could originate from at least two sources: First, the personal history of an individual could cause individuals to start at a different location in the information space. Different starting positions would lead to individual differences in learning because the path from less useful to more useful variables is different when the starting point (i.e., the less useful variable used in the beginning of learning) is different. Second, an important assertion of the direct learning approach is the existence of detectable information that specifies the path to follow to arrive at a more useful variable, which is called information for learning [32]. It seems reasonable to assume that individuals could differ in the capacity to pick up this information, hence differences in this capacity could be a source of individual differences. In short, the theory of direct learning provides some interesting leads to understand the origins of individual differences in learning an action. In the next paragraph we will turn to how this approach might be connected to other domains in the literature to reveal origins of individual differences.

The two origins of individual differences in the foregoing, that is, personal history and capacity to pick up information for learning, can be related to the literature on learning. Obviously, differences in individual motor experiences lay a foundation for the individual differences revealed here. One of the sources for differences in motor experiences is that individual differences are present very early in development, such as the development of reaching movements during infancy [11], [12]. The capacity to pick up information for learning might originate from common genetic variations [39], [40]. For instance, a common variation (Val66Met polymorphism) in the Brain-derived neurotrophic factor (BDNF), which is encoded by the BDNF gene, affects the anatomy of the hippocampus and prefrontal cortex. Thus, such genetic variation can induce changes in morphology of brain areas involved in learning and memory [41]. Moreover, this same variation in BDNF is thought to modulate possible synaptic changes in the motor cortex following a simple motor learning task [39]. Together this shows that understanding individual differences is required to achieve a full understanding of motor learning processes.

In order to understand individual differences, they have to be recognized and analysed. Individual differences are sometimes observed in the literature but their potential for understanding motor learning is often overlooked. For instance, Campola et al. [42] examined movement variability during a static pointing task performed with a tool showing that individuals differed in how variability was explored. In the same line, Cluff et al. [43] studied joint recruitment and coordination processes without focusing on individual differences when learning pole-balancing. When observing their data in reference to individual differences, results showed differences in joint configuration variability between participants. Thus, being open for possible individual differences when analyzing data can ensure a more accurate description of how participants acquire a novel task and can result in a deeper understanding of motor learning processes.

Considering our result of finding individual differences in a healthy adult population, future studies should examine individual differences in heterogeneous patient groups who are known to have difficulty with learning new motor skills, such as children with Developmental Coordination Disorder (DCD). Based on the results of the current study, it should be expected to find pronounced differences between patients, which is supported by the relatively high standard deviations in performance measures of tracing movements of DCD children compared to typically developed children and adults [44]. Understanding these differences would again improve our understanding of motor learning processes. Moreover, in rehabilitation, interventions focusing on enhancing motor skills should be tailored to the motor learning capacity and style of individual patients. This will help to better customize rehabilitation to the needs of patients and to improve its effect.

The second main finding of the current study is that the pronounced plateau phase in the grasping profile can be seen in all participants from the first trials onward. It seems that in tool grasping, the plateau phase is an integral component of the grasping profile. This is in line with literature also showing the existing of the plateau phase in the grasping profile of various tools [4], [23]–[27]. Currently, the origins of the plateau phase in tool prehension are far from being understood. It might well be that the existence of the plateau phase could be required in tool grasping because of the absence of proprioception. Because of the absence of the proprioceptive system, the control of movement occurs on the basis of the visual system only, which processes feedback slower than the proprioceptive system. The grasping network is said to operate on a very fast timescale [45]. Thus, it could be that the plateau phase emerges because of the slower processing of the visual information.

Following this suggestion, the slower processing speed of visual information could also explain why the plateau phase still exists towards the end of learning in most of the participants: First participants learned to rely on visual information only, thus the plateau phase decreased. It seems that the goal of learning is to resemble natural grasping. However, it could be possible that natural grasping cannot be exactly resembled with a pair of pliers, because it is inhibited by the slower processing speed of the visual system that will always result in a plateau phase. This supports the idea that the plateau phase is an integral component of tool grasping. This is in line with findings in the literature as Bouwsema et al. [4], [24] showed that even experienced prosthesis users showed a plateau phase in their grasping pattern.

Another finding was that different pliers with different transformation rules revealed different learning paths, that is, different changes in hand opening, plateau phase and hand closing between the Wider, Same, and Smaller pliers were observed. It should be noted that three groups with different participants were used whereby each group used a different pair of pliers. It could be that differences we found between the pliers were caused by differences between participants in the groups. However, participants were randomly divided into the three groups decreasing the likeliness of this possibility.

When learning to use a pair of pliers, participants had to adapt to the transformation assigned by the construction of the plier. The transformation of the Same Beak plier presupposed a one-to-one digit-beak mapping, whereas the transformation of the Wider Beak plier and the Smaller Beak plier presupposed that the digits-beak mapping is not one-to-one. Studies on tool transformation distinguish between compatible and incompatible tools [17], [18]. Incompatible tool transformations are defined as transformations where the direction of the movement of the hand does not correspond to the direction of the movement of the tool, which is in contrast to compatible tool transformations where the direction of both the movement of the hand and tool corresponds. This definition can also be conveyed to the current study, whereby the Same Beak plier reflected a compatible transformation and the Wider and Smaller Beak plier reflected an incompatible transformation. Beisert et al. [17] showed that tools incorporating a compatible transformation rule were handled faster and more accurately than tools with an incompatible transformation rule. This is in agreement with the current study as the mean duration of the grasping time for the Same Beak plier is shorter (268 ms) than the mean duration of grasping time of the Wider Beak plier (339 ms) and the Smaller Beak plier (355 ms). However, this does not help to explain the differences between the pliers, because learning paths of the Same Beak plier correspond more with the Smaller Beak plier than do the learning paths of the Smaller Beak plier with the ones of the Wider Beak plier.

In conclusion, the results of the current study showed individual learning paths when learning a novel discrete motor task. The motor learning differences we found stress the need for more individualized assessment of motor learning. Based on these findings we propose that individual differences play an important role in the understanding of motor learning and that individual differences should be considered more often in motor learning studies as well as in studies aiming to improve rehabilitation for patient groups.

## Supporting Information

Table S1 **Data of analysis variables of all individuals.**
(XLS)
